# P-1150. Staphylococcus aureus detection by direct plating and broth enrichment at 4 body sites in 921 adult inpatients and in nares of 377 healthcare workers

**DOI:** 10.1093/ofid/ofaf695.1344

**Published:** 2026-01-11

**Authors:** Maeve Hiehle, Carly Siciliano, Brooke M Talbot, Katrina Hofstetter, Leigh Cressman, Timothy D Read, Michael Z David

**Affiliations:** University of Pennsylvania, Philadelphia, Pennsylvania; University of Pennsylvania, Philadelphia, Pennsylvania; Emory University, Atlanta, Georgia; Emory University, Atlanta, Georgia; University of Pennsylvania Perelman School of Medicine, Philadelphia, Pennsylvania; Emory University School of Medicine, Atlanta, Georgia; University of Pennsylvania Perelman School of Medicine, Philadelphia, Pennsylvania

## Abstract

**Background:**

*Staphylococcus aureus* (SA) colonization detection at different anatomic sites by direct plating (DP) and overnight broth enrichment (BE) with quantitation of cultures is understudied.

**Methods:**

921 inpatients were cultured at several time points in 2 general hospital units in 12/2023 - 3/2025, with 6298 cultures at 4 body sites. Nares, throat and groin were tested by dry rayon swabs, and hands by phosphate buffered saline (PBS) wash. Also, 377 healthcare workers (HCW) had 601 nares cultures over 15 months. Swabs underwent DP on SA CHROMagar (SACA) (incubated at 37C for 24-48h) and then were placed in 5 mL tryptic soy broth (TSB) for BE. 45 uL of hand PBS was plated onto SACA, and 1 mL was added to 5 mL TSB for BE. If DP did not grow SA, 10 uL of TSB BE culture was plated on SACA and incubated 24-48h. SA cultures were quantified as ≥ 100 or < 100 colonies.

**Results:**

From all sites combined, 24.8% (1561/6298) of patient cultures grew SA; nares had 31% positive cultures, throat 24%, groin 17% and hand 28% (Table). 76.2% (1190/1561) SA were detected by DP and another 23.8% (371/1561) by BE. By body site, a greater percentage of SA cultures were detected by DP in the nares (88.5%) and throat (84.2%) and less in the groin (60.9%) and hand (65.2%).

In patient nares cultures, a greater percentage of plates had SA colony counts from BE that were ≥ 100 compared with DP (70% vs. 55%). This was also seen in the groin (43% vs. 25%) and hand (50% vs. 17%). In the throat, the opposite was found; colony counts from DP were higher, with 33% having ≥ 100 compared to BE (24%).

35% (210/601) of HCW nares cultures grew SA. Of 210 positives, 87% (182/210) were detected by DP and another 13% (28/210) by BE. Similar to patients, colony counts from BE (89%) were more often ≥ 100 than from DP (59%) (Table). HCW job type, month tested, season, and number of patient rooms entered on the day of culture were not significantly associated with SA isolation by DP vs. BE.

**Conclusion:**

HCW and patient nares SA cultures had similar results. DP cultures were more often positive from nares and throat than from groin and hand, suggesting a greater burden of SA colonization. Throat DP cultures growing SA had lower colony counts than nares, suggesting the throat may have a lower burden of SA colonization than the nares.

**Disclosures:**

All Authors: No reported disclosures

